# The burden of disease due to COVID-19 in Sweden: a disability-adjusted life years (DALY) study

**DOI:** 10.1093/eurpub/ckac131.568

**Published:** 2022-10-25

**Authors:** J Shedrawy, P Ernst, K Lönnroth, F Nyberg

**Affiliations:** 1 Department of Public Health Sciences, Karolinska Institutet, Stockholm, Sweden; 2 School of Public Health and Community Medicine, University of Gothenburg, Gothenburg, Sweden; 3 Centre for Epidemiology and Community Medicine, Stockholm County Council, Stockholm, Sweden

## Abstract

**Background:**

Many countries have been severely affected by the COVID-19 pandemic, including Sweden, which has been in the spotlight regarding its policies and their impact on mortality and morbidity. Therefore, it is of high interest to measure the disease burden of COVID-19 in terms of disability-adjusted life years (DALYs). DALYs have two main components: the years of life lost through premature death (YLL) and the number of years lived with disability (YLD). This study aims to measure DALYs due to COVID-19 in Sweden.

**Methods:**

This study used data from the nationwide multi-register observational study SCIFI-PEARL (Swedish COVID-19 Investigation for Future Insights - a Population Epidemiology Approach using Register Linkage) covering the entire Swedish population. The methodology used in the modelling and calculation of DALYs was based on the Global Burden of Disease guidelines, using Sweden-specific life tables for estimated life expectancies.

**Results:**

In Sweden, 152877 DALYs were lost to COVID-19 between March 2020 and October 2021, corresponding to 1447/ 100 000, 99,3% of which was attributed to YLL. DALYs loss occurred mainly among elderly groups with 66,78 % of DALYs being attributed to individuals above 70 years old. 57,6% of the lost DALYs occurred among men that lost more DALYs compared to females in all age groups.

**Conclusions:**

Similar to other countries, the burden of COVID-19 in Sweden is concentrated mainly among the elderly, which contributed to the highest DALY loss due to mortality. Yet, DALY loss remains lower for COVID-19 compared to other major non-communicable diseases such as cardiovascular diseases and neoplasms. The contribution of YLD was minimal. However, YLD due to post-Covid is not well understood and long-term disability is likely still underestimated.

**Key messages:**

• The burden of COVID-19 was mainly due to premature mortality in the older age groups.

• More research is needed especially on post-COVID disability to derive better estimates of YLD.

